# Effects of manual carotid compression in endovascular thrombectomy for acute anterior circulation large-vessel occlusion: a multicenter, propensity score-matching study

**DOI:** 10.3389/fneur.2025.1669778

**Published:** 2025-10-01

**Authors:** Li Bao, Shuang He

**Affiliations:** Department of Stroke Center, Affiliated Hospital of Nantong University, Nantong, China

**Keywords:** manual carotid compression, endovascular thrombectomy (EVT), large-vessel occlusion (LVO), propensity scorematching (PSM), acute ischemic stroke, balloon guide catheter (BGC)

## Abstract

**Background:**

Randomized controlled trials suggested that balloon guide catheters for proximal flow control (PFC) in endovascular thrombectomy (EVT) for acute anterior circulation large-vessel occlusion (LVO) have unsatisfactory results. Our study aimed to explore effects of manual carotid compression (MCC) achieving temporary PFC in EVT, without interfering with endovascular procedures.

**Methods:**

We retrospectively included 203 patients with acute occlusion of the intracranial internal carotid artery or the M1 or proximal M2 segments of the middle cerebral artery undergoing EVT from three independent centers (*n* = 80 in the MCC group and *n* = 123 in the non-MCC group). The primary outcome was the 90-day functional independence, defined as a modified Rankin Scale (mRS) score ≤ 2. Propensity score matching (PSM) analysis was conducted to compare outcomes.

**Results:**

In the overall cohort, the median age was 71 years (IQR 62–76), with 119 male patients (58.6%). Baseline characteristics between the two groups differed significantly in terms of hypertension (*p* = 0.017), previous stroke/TIA (*p* = 0.01), pre-stroke mRS (*p* = 0.003), baseline National Institutes of Health Stroke Scale (NHISS) score (*p* = 0.004), left occlusion (*p* < 0.001), and transfemoral access (*p* = 0.009). After adjusting for baseline characteristics using PSM, 34 matched pairs were analyzed. There was no significant difference in the 90-day functional independence between the two groups (44.1% vs. 32.4%, *p* = 0.454). The MCC group showed significantly lower mRS scores at 90 days (*p* = 0.031), with a higher proportion of patients scoring 0–1 (41.2% vs. 8.8%, *p* = 0.005). MCC significantly increased the first pass effect (FPE) rate (55.9% vs. 23.5%, *p* = 0.013) and the rate of modified Thrombolysis in Cerebral Infarction score ≥ 2b after the first pass (70.6% vs. 41.2%, *p* = 0.028), and reduced NHISS scores at 24 h after recanalization (*p* = 0.002) and at 7 days or discharge (*p* < 0.001). In terms of safety outcomes, MCC effectively reduced the incidence of intracranial hemorrhage (ICH) (14.7% vs. 47.1%, *p* = 0.009) and symptomatic ICH (sICH) (0% vs. 17.6%, *p* = 0.033).

**Conclusion:**

MCC in EVT for patients with acute anterior circulation LVO in our cohort could improve the 90-day mRS score and the proportion of patients with scores of 0–1, increase the reperfusion rate after the first pass and enhance early neurological improvement, while decreasing the incidence of ICH and sICH.

## Introduction

According to the estimated burden of stroke in China, among the population aged 40 and older, there were 3.4 million new stroke cases, 17.8 million individuals living with stroke, and 2.3 million stroke-related deaths in 2020 (1). Acute ischemic stroke (AIS) accounts for approximately 87% of strokes and has high rates of disability and mortality, bringing a huge burden to society (2, 3). Endovascular thrombectomy (EVT) has been proven effective in treating AIS caused by large vessel occlusion (LVO) in multiple randomized clinical trials (RCTs), establishing it as the standard treatment (4–7). The primary objective of EVT is to achieve rapid and complete reperfusion, ultimately resulting in favorable clinical outcomes. In this setting, several studies, including multicenter registry studies such as NASA and ROSSETTI, have found that transient proximal flow control (PFC) using a balloon guide catheter (BGC) was positively associated with improved EVT efficiency and better clinical outcomes in anterior circulation LVO (8–13). However, recent trials, including ProFATE and PROTECT-MT, have reported that the use of BGCs in EVT did not meet the expected results, and in some cases, led to poorer functional recovery (14–17). This may be related to challenges such as the incompatibility between BGCs and large-bore aspiration catheters, difficulties in achieving proper positioning of BGCs, and inadequate support for effective aspiration. Additionally, the advancements in first-line thrombectomy techniques and devices may also play a role (18). This raised the question: could extracorporeal PFC, when applied without interfering with endovascular procedures, improve EVT efficacy?

Manual carotid compression (MCC) is a non-invasive technique that effectively arrests anterior circulation blood flow. It has been recognized for its applications such as transcranial Doppler ultrasound, reducing brain embolism caused by arterial cannulation and aortic clamping, and treating arteriovenous fistulas (19–23). Given this, our multicenter study was aimed to investigate the impact of transient PFC via MCC on procedure and clinical outcomes in EVT-treated patients with anterior circulation LVO, focusing on functional independence at 90 days, the first-pass effect (FPE), the final recanalization quality, and distal/new region embolism. Propensity score matching (PSM) was employed to mitigate the confounding bias commonly encountered in observational studies.

## Methods

### Study design and patients

We retrospectively collected consecutive patients who underwent EVT treatment for acute occlusion of the intracranial internal carotid artery (ICA) or middle cerebral artery M1 or proximal M2 segments from November 2024 to March 2025 at three independent stroke centers. The exclusion criteria were as follows: (1) known or suspected pre-existing (chronic) large vessel occlusion in the symptomatic region; (2) intracranial hemorrhage (ICH) on CT prior to EVT; (3) tandem stenosis (70–99%) or occlusion; (4) coexisting intracranial tumors, aneurysms, intracranial infections, or arteriovenous malformations; (5) pre-stroke modified Rankin Scale (mRS) score > 2; (6) Alberta Stroke Program Early CT Score (ASPECTS) < 6; (7) Patients allergic to contrast agents. Patients were categorized into MCC or non-MCC group according to whether they received MCC in EVT. This study was conducted according to the Declaration of Helsinki with the approval of the Institutional Review Boards. Written informed consent was obtained from all patients or their legal representatives before inclusion.

### Data collection and outcomes

Baseline characteristics of the patients were comprehensively assessed, including age, sex, body mass index (BMI), medical history, pre-stroke mRS score and stroke characteristics involving the National Institutes of Health Stroke Scale (NIHSS) score [at admission, 24 h after recanalization, and 7 days after recanalization or at hospital discharge (whichever occurred first)], the admission ASPECTS, stroke etiology by TOAST criteria (24), and the occlusion site. Treatment details comprised the use of intravenous tissue plasminogen activator (IV tPA), the access approach (femoral or radial), general anesthesia, symptom onset to puncture time, procedural time (puncture to final successful recanalization time), MT technique [direct aspiration (DA) or combined DA + stent retriever (SR)].

The primary outcome was functional independence (defined as mRS score ≤ 2) at 90 days. Secondary outcomes included mRS score at 90 days, other dichotomous analysis of score on the mRS at 90 days (0–1 vs. 2–6, 0–3 vs. 4–6, 0–4 vs. 5–6, and 0–5 vs. 6); change of the NHISS score from baseline to 24 h after recanalization, and to 7 days after treatment or at hospital discharge (whichever occurred first); the final recanalization quality assessed by the modified Thrombolysis in Cerebral Infarction (mTICI) score at the final intracranial angiogram; FPE (defined as an mTICI = 3 after the first pass); mTICI score ≥ 2b after the first pass; the number of passes attempts to achieve successful recanalization (no more than 4); occurrence of emboli in a new or distal territory. Safety outcomes included all-cause mortality at 90 days after treatment; all ICH and symptomatic intracranial hemorrhages (sICH) according to the Heidelberg criteria (25). Neurological improvement was defined as the reduction of the NHISS score.

### Procedure

EVT was performed by experienced neurointerventionists following the current guidelines. DA or combined DA + SR were considered the first-line thrombectomy technique. The coaxial or exchange technique was used to advance a long sheath to the highest possible position in the internal carotid artery, with the aspiration catheter or distal access catheter positioned as close as possible to the site of occlusion. The selection of any endovascular devices, including the long sheath (Ballast, Balt, France), aspiration catheter (Ace, Penumbra, USA; React, Medtronic, USA), distal access catheter (Zenith, China; Soft, TONBRIDGE, China), microcatheter (rebar, Medtronic, USA), microguidewire (Synchro SELECT, Stryker, USA), and stent retrievers (Solitaire; Medtronic, USA; EmboTrap, Cerenovus, USA), was determined at the discretion of the operators. In the MCC group, the assistant synchronously acupressured the ipsilateral carotid artery during negative pressure aspiration or stent retraction, and in the non-MCC group, EVT was done conventionally without extra intervention. The assistant ensured the forward blood flow arrest by applying ultrasound at the compression site. The choice of anesthesia (general anesthesia or conscious sedation), the change of thrombectomy strategy or devices, and any resuscitation treatments, including intracranial angioplasty with or without stenting and intra-arterial drug therapy, were determined by the operator, considering the occlusion mechanism, the patient’s clinical condition, and other relevant factors. Strict systolic blood pressure management should be applied to all patients following EVT (≤160 mmHg for those without hemorrhagic transformation immediately post-procedure, ≤140 mmHg for those with hemorrhagic transformation, and all should not be lower than 120 mmHg). All patients should undergo Non-Contrast CT within 24 h after treatment; if necessary, additional imaging should be performed at any time in case of neurological deterioration.

### Statistical analysis

All statistical analysis was conducted using RStudio software (version 4.4.3). Graphs were created using GraphPad version 10.1.2. The normality of continuous variables was assessed using the Kolmogorov–Smirnov test. Continuous variables with normal distribution were expressed as means (SD), while non-normally distributed variables were presented as medians (IQR). Categorical variables were reported as frequencies (proportions). Comparisons of continuous variables were performed using the t-test or Mann–Whitney U test. Categorical variables were compared using the *χ*^2^ test or Fisher’s exact test. PSM analysis was performed on primary and secondary outcomes using the nearest-neighbor method to adjust for potential confounders between MCC and non-MCC groups. A 1:1 matching without replacement was performed based on a caliper of 0.1. Covariate balance between matched groups was assessed using *p*-values and standardized mean differences (SMD). The factors adjusted for in the PSM analysis included age, sex, BMI, medical history, stroke characteristics, IV-tPA, transfemoral access, general anesthesia, symptom onset to puncture time, first-line technique. A two-tailed *p* value of <0.05 was considered statistically significant.

## Results

### Baseline characteristics and outcomes before PSM

**Figure 1:**
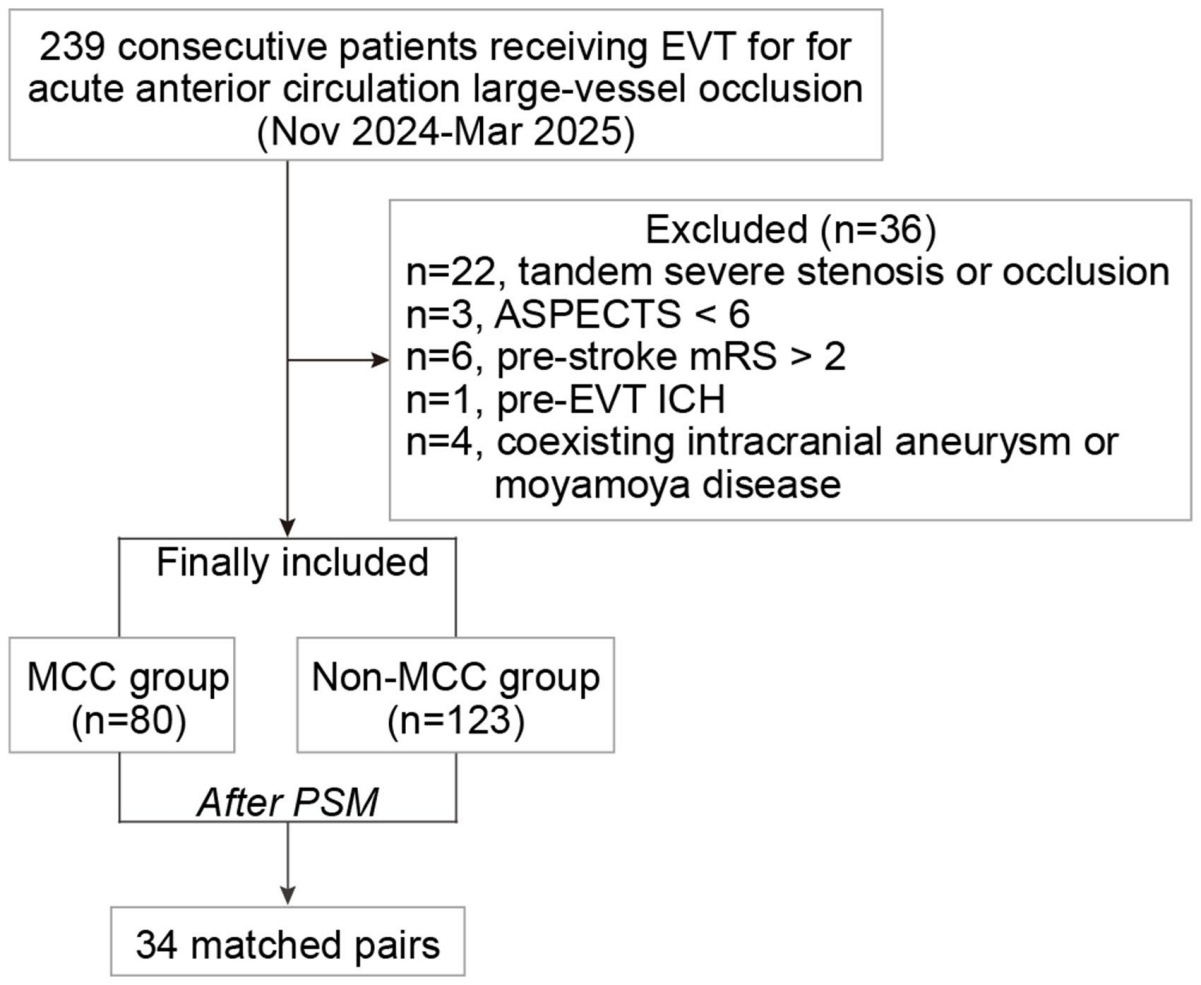
Flowchart. EVT, Endovascular thrombectomy; ASPECTS, Alberta Stroke Program Early CT Score; mRS, modified Rankin Scale; ICH, intracranial hemorrhage; MCC, manual carotid compression; PSM, propensity score matching.

Between November 2024 and March 2025, 239 AIS patients receiving MCC in EVT were assessed for eligibility, of whom 36 were excluded, and 203 were ultimately included (*n* = 80 in the MCC group and *n* = 123 in the non-MCC group) (Figure 1). In the overall cohort, the median age was 71 years (IQR 62–76), with 119 male patients (58.6%). The median baseline NHISS score was 15 (IQR 12–20), and the median baseline ASPECTS was 9 (IQR 8–10). The median time from symptom onset to puncture was 469 min (IQR 330–570). A total of 55patients (27.1%) received IV tPA prior to EVT. The occlusion sites were intracranial ICA [56 (27.6%)], M1 [122 (60.1%)], and M2 [25 (12.3%)]. According to the TOAST classification, 77 patients (37.9%) were identified as having atherosclerosis-related LVO, and 80 patients (39.4%) were judged to have cardiogenic embolism.

There were significant differences between the two groups in terms of hypertension (*p* = 0.017), previous stroke/TIA (*p* = 0.01), pre-stroke mRS (*p* = 0.003), baseline NHISS (*p* = 0.004), left occlusion (*p* < 0.001), and transfemoral access (*p* = 0.009) (Table 1). No significant difference was observed between the groups regarding the first-line thrombectomy technique (*p* = 0.972). Four patients (5%) in the MCC group and five patients (4.1%) in the non-MCC group received intra-arterial 5% tirofiban injection. Additionally, 6 patients (7.5%) in the MCC group and 11 patients (8.9%) in the non-MCC group underwent stent implantation as a rescue measure.

**Table 1:** Comparison of baseline characteristics before and after propensity score matching.

Variables	Before PSM				After PSM			
	MCC (*n* = 80)	Non-MCC (*n* = 123)	*p*	SMD	MCC (*n* = 34)	Non-MCC (*n* = 34)	*p*	SMD
Age (years)	70.5 (56.3–76.8)	71 (66–75)	0.855	0.111	70 (58–74.3)	71 (53–76)	0.833	0.106
Female	30 (37.5%)	54 (43.9%)	0.448	0.131	14 (41.2%)	10 (29.4%)	0.446	0.248
BMI (kg/m^2^)	25.2 (21.1–27.3)	23.9 (21.5–26.8)	0.098	0.264	24.5 (21.2–26.5)	24 (21.3–27.4)	0.954	0.070
Medical history								
Hypertension	54 (67.5%)	102 (82.9%)	0.017^*^	0.363	25 (73.5%)	21 (61.8)	0.437	0.253
Diabetes	24 (30.0%)	30 (24.4%)	0.471	0.126	10 (29.4)	12 (35.3)	0.795	0.126
Atrial fibrillation	36 (45.0%)	45 (36.6%)	0.294	0.172	14 (41.2%)	9 (26.5%)	0.305	0.315
Coronary heart disease	16 (20.0%)	27 (22.0%)	0.875	0.048	5 (14.7%)	2 (5.9%)	0.425	0.293
Dyslipidemia	26 (32.5%)	30 (24.4%)	0.270	0.180	8 (23.5%)	7 (20.6%)	>0.999	0.071
Previous stroke/TIA	22 (27.5%)	15 (12.2%)	0.010^*^	0.391	3 (8.8%)	7 (20.6%)	0.304	0.337
Pre-stroke mRS	0 (0–1)	0 (0–0)	0.003^*^	0.365	0 (0–1)	0 (0–0)	0.399	0.143
Stroke characteristics								
Baseline NHISS	14 (12–18.8)	17 (13–20)	0.004^*^	0.408	15 (12–18.5)	15 (13–19)	0.944	0.112
Baseline ASPECTS	9 (8–10)	9 (8–10)	0.493	0.092	10 (8–10)	10 (9–10)	0.565	0.233
Etiology by TOAST			0.301	0.223			0.415	0.326
Atherosclerosis	26 (32.5%)	51 (41.5%)			13 (38.2%)	15 (44.1%)		
Cardioembolism	32 (40.0%)	48 (39.0%)			14 (41.2%)	9 (26.5%)		
Undetermined or other reason	22 (27.5%)	24 (19.5%)			7 (20.6%)	10 (29.4%)		
Occlusion site			0.798	0.097			0.931	0.092
Intracranial ICA	20 (25.0%)	36 (29.3%)			9 (26.5%)	10 (29.4%)		
M1	40 (62.5%)	72 (58.54%)			19 (55.9%)	19 (55.9%)		
M2	10 (12.5%)	15 (12.2%)			6 (17.6%)	5 (14.7%)		
Left occlusion	60 (75%)	54 (43.9%)	<0.001^*^	0.668	22 (64.7%)	20 (58.8%)	0.803	0.121
Treatment details								
IV tPA	22 (27.5%)	33 (26.8%)	>0.999	0.015	10 (29.4%)	9 (26.5%)	>0.999	0.066
Transfemoral access	78 (97.5%)	105 (85.4%)	0.009^*^	0.444	32 (94.1%)	27 (79.4%)	0.152	0.445
General anesthesia	74 (92.5%)	111 (90.2%)	0.764	0.080	30 (88.2%)	31 (91.2%)	>0.999	0.097
Symptom onset to puncture time (min)	477.5 (312–570)	454 (336–540)	0.609	0.014	540 (337.3–585)	454 (337–663.3)	0.663	0.014
Procedural time (min)	75.5 (50.5–100)	84 (48–97)	0.890	0.019	67.5 (50–91)	83 (49–90.3)	0.458	0.200
First-line technique			0.972	0.026			0.467	0.237
DA	38 (47.5%)	60 (48.8%)			15 (44.1%)	19 (55.9%)		
Combined DA + SR	42 (52.5%)	63 (51.2%)			19 (55.9%)	15 (44.1%)		

Values were shown as median (IQR) or frequency (%). ^*^Statistical significance. PSM, propensity score matching. MCC, manual carotid compression; BMI, body mass index; TIA, transient ischemic attack; mRS, modified Rankin Scale; NHISS, National Institutes of Health Stroke Scale; ASPECTS, Alberta Stroke Program Early CT Score; ICA, internal carotid artery; IV tPA intravenous tissue plasminogen activator; DA, direct aspiration; SR, stent retriever; SMD, standardized mean difference.

**Figure 2:**
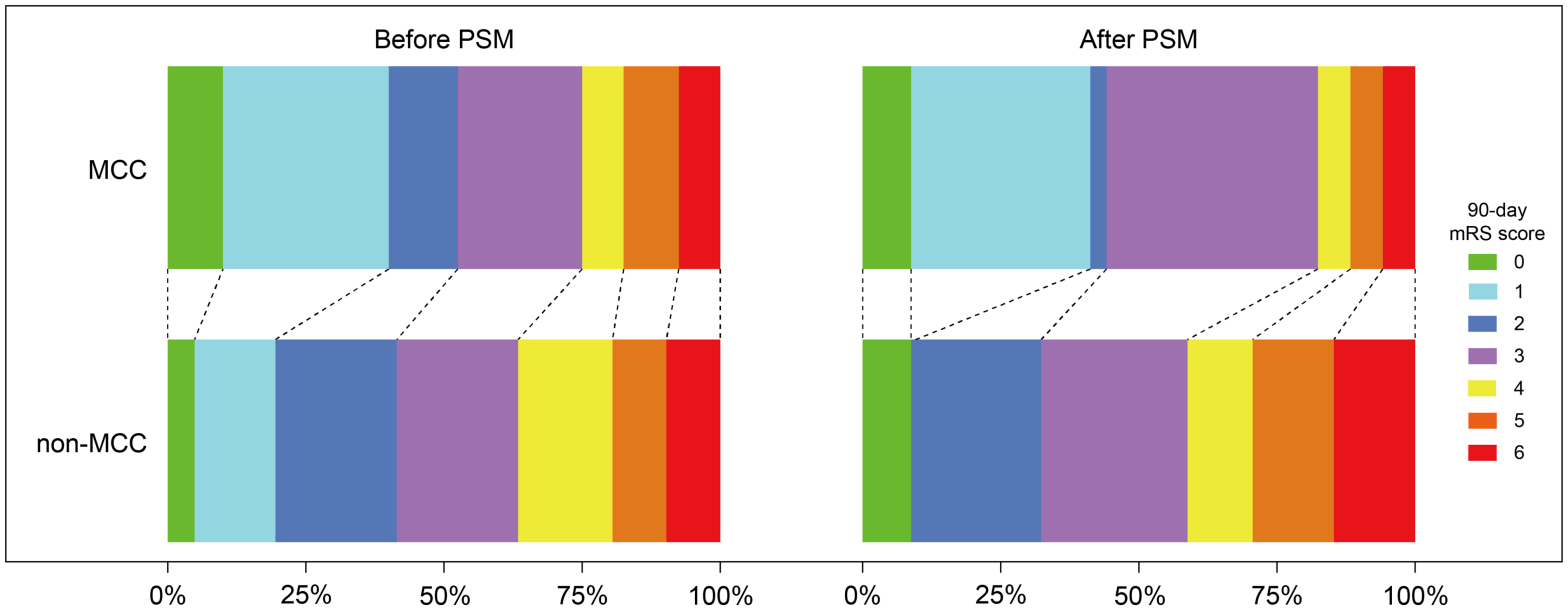
Distribution of the modified Rankin Scale (mRS) at 90 days of patients with anterior circulation large-vessel occlusion treated with or without manual carotid compression (MCC) in endovascular thrombectomy before and after propensity score matching (PSM).

There was no significant difference between the two groups in the primary outcome of functional independence at 90 days (52.5% vs. 41.5%, *p* = 0.162) (Table 2). There was significant difference between the two groups in the 90-day mRS score [2 (1–3.8) vs. 3 (2–4), *p* = 0.021] and the predefined binary comparison of 0–1 (vs 2–6) (40% vs. 19.5%, *p* = 0.002). The distribution of the 90-day mRS scores for both groups was shown in Figure 2. The MCC group had a significantly higher PFE rate (50% vs. 29.3%, *p* = 0.005) and mTICI≥2b after the first pass rate (60% vs. 36.6%, *p* = 0.002). The MCC group also demonstrated significantly better neurological improvement (change of NIHSS score) at 24 h post-reperfusion (*p* < 0.001), compared to the non-MCC group. However, there was no significant difference in neurological improvement between the two groups at 7 days or at discharge (*p* = 0.383). Regarding safety outcomes, patients who received MCC had a lower incidence of ICH (27.5% vs. 58.5%; *p* < 0.001) and sICH (5% vs. 22%; *p* = 0.002). There was no difference between the two groups in the mortality at 90 days and other secondary outcomes. The causes of death in the MCC group included three cases of large intracranial hematoma, one case of malignant brain edema, and two cases of severe infection. In the non-MCC group, the causes of death included nine cases of large intracranial hematoma, two cases of malignant brain edema and one case of post-discharge pulmonary infection.

**Table 2:** Outcomes before and after propensity score matching.

Outcomes	Before PSM				After PSM			
	MCC (*n* = 80)	Non-MCC (*n* = 123)	*p*	SMD	MCC (*n* = 34)	Non-MCC (*n* = 34)	*p*	SMD
Primary outcomes								
Functional independence at 90 d	42 (52.5%)	51 (41.5%)	0.162	0.222	15 (44.1%)	11 (32.4%)	0.454	0.244
Secondary outcomes								
Dichotomized mRS scores at 90 d								
mRS score at 90 d	2 (1–3.8)	3 (2–4)	0.021^*^	0.307	3 (1–3)	3 (2–5)	0.031^*^	0.558
0–1 (vs 2–6)	32 (40.0%)	24 (19.5%)	0.002^*^	0.460	14 (41.2%)	3 (8.8%)	0.005^*^	0.805
0–3 (vs 4–6)	60 (75.0%)	78 (63.4%)	0.115	0.253	28 (82.4%)	20 (58.8%)	0.062	0.535
0–4 (vs 5–6)	66 (82.5%)	99 (80.5%)	0.861	0.052	30 (88.2%)	24 (70.6%)	0.134	0.447
0–5 (vs 6)	74 (92.5%)	111 (90.2%)	0.764	0.080	32 (94.1%)	29 (85.3%)	0.425	0.293
Final mTICI ≥ 2b	74 (92.5%)	114 (92.7%)	>0.999	0.007	30 (88.2%)	29 (85.3%)	>0.999	0.087
Final mTICI = 3	58 (72.5%)	78 (63.4%)	0.233	0.196	21 (61.8%)	19 (55.9%)	0.805	0.120
mTICI≥2b after the first pass	48 (60.0%)	45 (36.6%)	0.002^*^	0.482	24 (70.6%)	14 (41.2%)	0.028^*^	0.620
mTICI = 3 after the first pass (FPE)	40 (50.0%)	36 (29.3%)	0.005^*^	0.434	19 (55.9%)	8 (23.5%)	0.013^*^	0.701
No. of passes	1 (1–3)	2 (1–3)	0.026^*^	0.226	1 (1–2)	2 (1–2)	0.062	0.306
Change of NIHSS score								
24 h after recanalization	−3.5 (−5 to −2)	−2 (−5 to 0)	<0.001^*^	0.312	−4 (−5 to −2)	−1 (−4 to 4)	0.002^*^	0.780
7 days or at discharge	−7.5 (−10.8 to −4.3)	−7 (−10 to −3)	0.383	0.136	−9.5 (−12 to −6)	−6 (−8 to −3)	<0.001^*^	0.677
Emboli to new territory	2 (2.5%)	6 (4.9%)	0.630	0.126	2 (5.9%)	0	0.473	0.354
Emboli to distal territory	12 (15.0%)	21 (17.1%)	0.844	0.057	4 (11.8%)	10 (29.4%)	0.134	0.447
Safety outcomes								
ICH	22 (27.5%)	72 (58.5%)	<0.001^*^	0.660	5 (14.7%)	16 (47.1%)	0.009^*^	0.748
sICH	4 (5.0%)	27 (22.0%)	0.002^*^	0.512	0	6 (17.6%)	0.033^*^	0.655
Mortality at 90 d	6 (7.5%)	12 (9.8%)	0.764	0.080	2 (5.9%)	5 (14.7%)	0.425	0.293

Values were shown as median (IQR) or frequency (%). ^*^Statistical significance. PSM, propensity score matching; MCC, manual carotid compression; mRS, modified Rankin Scale; mTICI, modified Thrombolysis in Cerebral Infarction; FPE, first-pass effect; NHISS, National Institutes of Health Stroke Scale; ICH, intracranial hemorrhages; sICH, symptomatic intracranial hemorrhages; SMD, standardized mean difference.

### Propensity score matching analysis

After adjusting for differences in age, sex, BMI, hypertension, diabetes, atrial fibrillation, coronary heart disease, dyslipidemia, previous stroke/TIA, pre-stroke mRS score, NHISS at admission, baseline ASPECTS, etiology by TOAST, occlusion site, left occlusion, IV-tPA, transfemoral access, general anesthesia, symptom onset to puncture time, and first-line technique, 34 pairs of MCC and non-MCC treatments were successfully matched after PSM analysis. After matching, the baseline characteristics of the two groups were balanced (Table 1). After PSM analysis, there was no significant difference in functional independence at 90 days between the two groups (44.1% vs. 32.4%, *p* = 0.454). However, the MCC group had a significantly lower mRS score at 90 days (*p* = 0.031), primarily due to a higher proportion of patients with scores of 0–1 (41.2% vs. 8.8%, *p* = 0.005) (Table 2; Figure 2). Additionally, the MCC group showed significantly higher FPE rates (55.9% vs. 23.5%, *p* = 0.013) and first-pass mTICI ≥2b rates (70.6% vs. 41.2%, *p* = 0.028). Patients in the MCC group had significantly greater improvement in NHISS scores at 24 h after recanalization (*p* = 0.002) and at 7 days or discharge (*p* < 0.001) compared to the non-MCC group. In terms of safety outcomes, MCC significantly reduced the occurrence of ICH (14.7% vs. 47.1%, *p* = 0.009) and sICH (0% vs. 17.6%, *p* = 0.033).

## Discussion

In this multicenter PSM study, we found that temporary PFC achieved by MCC in EVT did not significantly improve the primary outcome (90-day functional independence) in patients with acute anterior circulation LVO. The functional, imaging, and safety outcomes in the non-MCC group of our study were similar to the control groups in the ProFATE and PROTECT-MT studies (15, 16). However, in terms of secondary outcomes, after controlling for confounding factors with PSM analysis, we observed that MCC significantly improved the 90-day mRS score (mainly reflected in the higher proportion of 0–1 scores), PFE rate, first-pass mTICI ≥2b rate, early neurological improvement (change in NIHSS score), and the rates of ICH and sICH.

In recent years, there has been a focus on optimizing techniques and strategies to improve angiographic and clinical outcomes in EVT. Currently, the use of BGCs, which provide proximal flow arrest, has become a contentious issue in first-line thrombectomy approaches for AIS patients with anterior circulation LVO. Contrary to previous observational studies, the PROTECT-MT trial found that the use of BGCs was associated with poorer functional recovery and procedure results, as well as a higher rate of ICA vasospasm (15). As analyzed by Liu et al. (15) and Dhillon et al. (18), on one hand, the rapid advancement of thrombectomy techniques and devices has leveled out the benefits of BGCs in earlier years; on the other hand, BGCs has limitations such as restricting the use of large-bore aspiration catheters, affecting the stability of the access, and reducing collateral circulation flow. The disappointing results of BGCs in EVT led us to propose MCC, a technique that arrested anterior blood flow ex vivo without disrupting the endovascular procedure.

The MCC group had a significantly higher proportion of patients with minimal functional impairment (mRS scores 0–1), and early stroke severity (NHISS change) was significantly reduced with higher FPE rates and first-pass mTICI ≥2b rates, suggesting that MCC’s improvement in thrombectomy efficiency was more likely to facilitate the transition of patients from mild disability to complete independence. Studies have shown that PFE is associated with better clinical outcomes and is a key goal of EVT in achieving rapid and complete reperfusion (26, 27). Achieving FPE can effectively reduce the risks of thrombus rupture and distal migration, both of which are closely associated with poor outcomes (28–30), although the reduction in embolization rate in the new/distal territory did not reach statistical significance in our study. However, in this study, MCC did not significantly reduce the proportion of patients with an mRS score ≥2. This might be attributed to MCC’s more proximal blood flow arrest, which reduced more backpressure from ipsilateral collateral circulation that aided in thrombectomy (31) and limited the maintenance of the ischemic penumbra. Additionally, we observed that MCC led to a median improvement of 3 points in the early NHISS score compared to Non-MCC. The NHISS score reflects the severity of the stroke, and while a 3-point reduction may not significantly improve function in patients with more severe strokes (mRS ≥ 3), it can be sufficient to reverse functional impairments in those with mild deficits, improving the mRS score from 2 to 0 or 1. Therefore, the analysis of limited data from this exploratory study suggests that MCC has the potential for clinical benefits, as indicated by the improvement in secondary outcomes (proportion of patients with an mRS score of 0–1). Moreu et al. (32) found that positioning BGCs higher was associated with better reperfusion rates and improved clinical outcomes when used with SR technology alone. Therefore, expanding the sample size of the MCC study and further exploring the optimal site of PFC are valuable for optimizing EVT efficacy in treating anterior circulation LVO.

Regarding safety outcomes, the MCC group showed a significant reduction in the incidence of ICH and sICH before and after PSM analysis. Enhanced first-pass reperfusion efficiency minimized mechanical damage to the blood–brain barrier at the occlusion site and ischemic time. Additionally, in AIS patients with high blood pressure and significant blood pressure variability, the risk of ICH after EVT is increased (33, 34). Early control of hemodynamic impact after reperfusion can mitigate reperfusion injury and promote recovery of the ischemic penumbra tissue (35). Similarly, the study by Deng et al. (36) on rapid local ischemic postconditioning, which reduced reperfusion injury and promoted functional independence, is also based on this theory. During thrombectomy, MCC has the value of reducing transient blood flow impact upon reperfusion, thereby providing neuroprotection. Therefore, theoretically, regular MCC after reperfusion may also hold potential neuroprotective applications for ischemic postconditioning. Xu et al. (37) also highlighted the role of non-pharmacological therapies in improving the imbalance caused by hyperperfusion during the reperfusion phase. Our study considered MCC as an efficient and effective non-pharmacological approach. Therefore, our research suggested that MCC warrants further large-scale and in-depth studies to confirm its value in preventing hemorrhagic transformation associated with endovascular treatments.

MCC, a straightforward approach, offers the potential to significantly improve clinical outcomes through its benefits, including reduced costs, shorter procedure times, and minimal risks. The RCT by Hillebrand et al. (23) and the *in vitro* simulation by Isingoma et al. (19) showed that MCC could effectively reduce the rate of cerebral embolism during arterial catheterization and aortic clamping. Furthermore, MCC provides a safe and effective treatment alternative for patients with arteriovenous fistulas who are ineligible for endovascular therapy (22, 38). Our study aimed to investigate the effect of PFC without interfering with endovascular procedures, and MCC perfectly meeted this intervention requirement. A key advantage of this trial is its practicality and generalizability. Unlike RCTs involving BGCs, there were no specific requirements or limitations regarding the access and devices for both groups. Moreover, the intervention poses no cost and relatively less endothelial damage. Therefore, this study demonstrated the feasibility and effectiveness of MCC in EVT, offering potential for optimizing future thrombectomy strategies to improve outcomes. Furthermore, this study validated the benefits of temporary PFC and provided a foundation and insights for advancing PFC devices, including BGCs. In general, MCC or optimized BGC, through PFC, can serve as an adjunctive strategy in various scenarios, such as preventing plaque dislodgement during the treatment of atherosclerotic disease, reducing cyanoacrylates glue reflux during the treatment of arteriovenous malformations, and promptly reducing flow in cases of hemorrhagic complications following endovascular procedure.

This study has some limitations. First, despite our efforts to control for confounders using PSM, the retrospective nature of this study inherently introduced unavoidable selection bias and unmeasured confounders. Additionally, the relatively small number of matched pairs after PSM may affect the reliability of the exploratory research results. Thirdly, the lack of a detailed assessment of the impact of collateral circulation and the Willis compensation on the efficacy of MCC may undermine the stability of the positive outcome generalization. Fourthly, the whole trial cohort consisted of Chinese patients. A large proportion (37.9%) of AIS patients with an atherosclerotic etiology rely more on long-established collateral circulation. Fifthly, since the operators in this study inevitably knew the treatment intervention, this could have influenced the management of patients during and after EVT. Sixthly, our patients received either DA or combined DA + SR techniques, and our results may not be generalizable apply to patients who received SR alone.

## Conclusion

In AIS patients with anterior circulation LVO in our cohort, the use of MCC during EVT to achieve transient PFC could improve the proportion of participants with a 90-day mRS score of 0–1, FPE rates, first-pass mTICI ≥2b rates, and change in NIHSS score, and decreased the incidence of ICH and sICH. To further confirm the efficacy and safety of MCC in EVT for AIS, larger-scale, multi-center RCTs are necessary.

## Data Availability

The raw data supporting the conclusions of this article will be made available by the authors, without undue reservation.
